# Pharmacist-coordinated IDST treatment bundle achievement and impact on mortality in MRSA bacteraemia: a single-centre retrospective cohort study

**DOI:** 10.1016/j.infpip.2026.100578

**Published:** 2026-08-08

**Authors:** Aiju Endo, Yuki Hanai, Takahiro Mikawa, Daiki Asakawa, Taiki Ishibe, Yu Nakane, Misako Tanaka, Daichi Mitsui, Shinji Kido, Takeshi Nonaka, Ken Fujimori, Nozomi Kudo, Yuta Nakadoi, Yukimasa Kobayashi, Rintaro Negishi, Ayaka Fujimori, Mika Takatori, Yasuyuki Natsume, Kaori Matsumoto, Kazuaki Matsumoto

**Affiliations:** a Department of Pharmacy, Yamanashi Prefectural Central Hospital, Kofu-city, Yamanashi, Japan; b Laboratory of Clinical Infectious Diseases and Therapeutics, Meiji Pharmaceutical University, Kiyose-city, Tokyo, Japan; c Department of General Medicine and Infectious Diseases, Yamanashi Prefectural Central Hospital, Kofu-city, Yamanashi, Japan; d Internal Medicine, Ehime Prefectural Minamiuwa Hospital, Ainan-cho, Ehime, Japan; e Internal Medicine, Tsuru Municipal General Hospital, Tsuru-city, Yamanashi, Japan; f Hematology and Oncology, Kameda Medical Center, Kamogawa, Chiba, Japan; g Infection Control Department, Yamanashi Prefectural Central Hospital, Kofu-city, Yamanashi, Japan; h Division of Pharmacodynamics, Keio University Faculty of Pharmacy, Tokyo, Japan

**Keywords:** Methicillin-resistant *Staphylococcus aureus* (MRSA) bloodstream infection, Pharmacist-coordinated, Antimicrobial stewardship, Infectious disease support, Bundle, Quality indicator (QI)

## Abstract

**Background:**

Methicillin-resistant *Staphylococcus aureus* (MRSA) bacteraemia carries high mortality. At Yamanashi Prefectural Central Hospital, antimicrobial stewardship has focused on appropriate drug use. In 2022, a pharmacist-coordinated Infectious Disease Support Team (IDST) was established to optimize management, including diagnostics, dosing, and treatment duration. We evaluated the effects of IDST participation on the adherence to a predefined *Staphylococcus aureus* bacteraemia treatment bundle and mortality.

**Methods:**

We conducted a single-centre retrospective cohort study of adults with MRSA bacteraemia diagnosed between April 2020 and June 2025. Patients were categorized into IDST intervention or non-intervention groups. Bundle adherence (≥ 75% defined as ≥ 9/12 items) and 28- and 90-day mortality were analysed using Kaplan–Meier analysis with log-rank tests and Cox proportional-hazards models; two-sided *P* < 0.05 was considered significant.

**Results:**

Eighty-five patients were included in the present study. Overall, bundle adherence ≥75% occurred in 57.6%; adherence was higher with IDST than without (79.5% vs 39.1%); 28-day mortality occurred in 26 (30.6%) and 90-day mortality in 42 (49.4%), respectively. Survival analysis showed lower 28-day (log-rank *P*=0.031) and 90-day mortality (*P*=0.017) with IDST. In multi-variable models, IDST remained independently protective at 28 days (hazard ratio [HR] 0.328, 95% CI: 0.136–0.792; *P*=0.013) and 90 days (HR 0.427, 95% CI: 0.215–0.846; *P*=0.015).

**Conclusions:**

Pharmacist-coordinated IDST co-management significantly improved adherence to evidence-based care and was associated with reduced short- and intermediate-term mortality in patients with MRSA bacteraemia. This model may be applicable to hospitals with limited continuous on-site availability of infectious disease physicians, provided that specialist oversight remains accessible.

## Introduction

*Staphylococcus aureus* bacteraemia (SAB) is a severe infection caused by methicillin-resistant *Staphylococcus aureus* (MRSA), with limited treatment options primarily including vancomycin (VCM) and daptomycin [1,2]. MRSA bacteraemia has a poor prognosis, with a reported 28–30-day mortality rate of 30–50%, a secondary focus rate (e.g. infective endocarditis [IE]) of approximately 20%, progression to septic shock in 10–40% of cases, and recurrence of approximately 9% [[3], [4], [5], [6], [7], [8]]. Accordingly, when a complicated infection is present, antimicrobial therapy alone is often insufficient; comprehensive management that integrates early diagnosis and appropriate source control is recommended [[9], [10], [11], [12]]. According to these principles, the Infectious Diseases Society of America recommends following a SAB treatment bundle to ensure appropriate diagnosis and therapy [13].

Yamanashi Prefectural Central Hospital previously relied on a pharmacist-led Antimicrobial Stewardship Team (AST) to support primary services. In 2022, the hospital established an Infectious Disease Support Team (IDST) to provide comprehensive, proactive support across the continuum of infectious disease (ID) care. The IDST, comprising physicians, pharmacists, and nurses, actively intervenes in bacteraemia management by proposing antimicrobial selection, dosing, and duration, recommending diagnostic tests and procedures for source control, and continuing joint consultation until treatment stabilizes or a clear prognosis is established. The key distinction from the previous AST framework was that the IDST shifts the focus from the appropriate use of antimicrobial agents to the treatment of infectious diseases and linked antimicrobial stewardship with SAB-specific care processes, including diagnostic evaluation, source-control assessment, and review of treatment duration, under specialist oversight.

As access to ID specialists is limited, pharmacists assume task-shifting responsibilities. The pharmacists consistently followed the following workflow: selecting patients for intervention, conducting active consultations, summarizing key points through chart review and team presentations, performing bedside physical assessments, documenting diagnostic assistance and recording it in the electronic medical record, and providing feedback and treatment recommendations to the attending physician. This approach supplements the constrained physician resources and aims to improve the quality of care.

Multiple reports have indicated that SAB treatment bundles and adherence to quality indicators (QIs) improve care processes and outcomes [[14], [15], [16], [17], [18], [19], [20], [21]]. Pharmacist-driven SAB initiatives, pharmacist-directed stewardship protocols, and ID consultation or stewardship co-management models have also been reported [[22], [23], [24], [25]]. However, evidence remains limited regarding pharmacist-coordinated IDST co-management that combines task-shifted pharmacist coordination, bedside assessment, specialist oversight, and SAB bundle implementation for MRSA bacteraemia. Therefore, we examined the effect of the pharmacist-coordinated IDST intervention at our hospital on bundle adherence and mortality among patients with MRSA bacteraemia.

## Methods

### Study design

This single-centre retrospective cohort study was conducted at the Yamanashi Prefectural Central Hospital and enrolled patients with MRSA detected in one or more sets of blood cultures between April 2020 and June 2025. The exclusion criteria were as follows: (1) death within 48 h of confirmation of blood culture positivity; (2) receipt of VCM or teicoplanin (TEIC) without therapeutic drug monitoring (TDM); and (3) receipt of VCM in whom population-based area under the blood concentration–time curve (AUC) prediction was not feasible (e.g. neonates, patients undergoing continuous renal replacement therapy).

### Infectious disease support team (IDST) intervention

**Figure 1:**
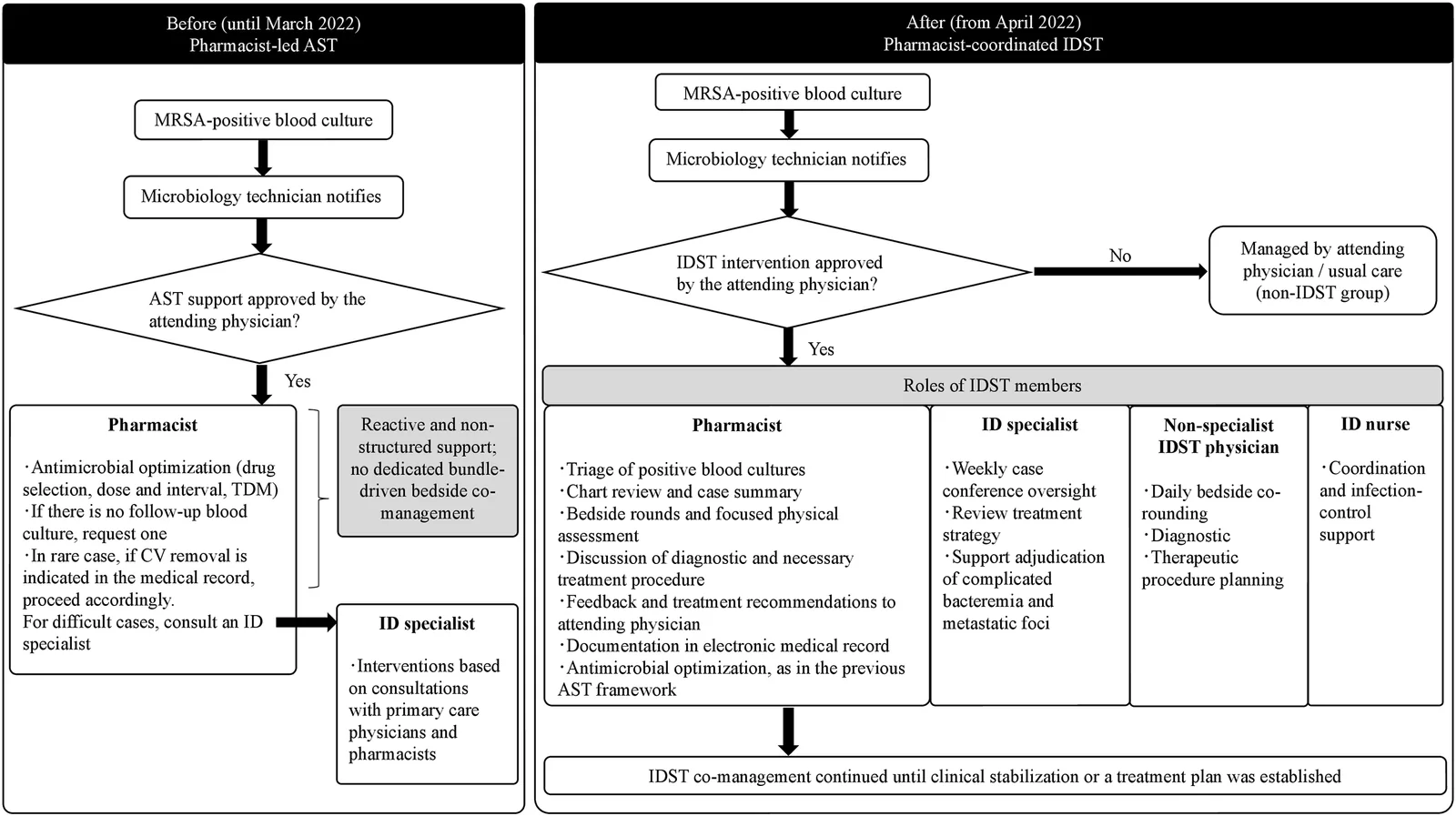
Consultation structures before and after IDST activities. IDST, Infectious Disease Support Team.

The IDST, composed of pharmacists, physicians, including ID specialists and non-specialist physicians in the Division of Infectious Diseases, and an ID nurse, provided proactive co-management with the attending physicians (Figure 1). Microbiology laboratory technologists were not formal members of the IDST; their role was to notify the IDST pharmacist of positive blood culture results. In this programme, ‘pharmacist-coordinated’’ indicates that pharmacists served as the initial coordinator and practical driver of the IDST workflow, rather than replacing physician decision-making.

All patients with MRSA-positive blood cultures were screened by the IDST pharmacist after notification from the microbiology laboratory. In principle, approval for joint consultation was requested from the attending physician for all eligible patients, except for patients already managed by the Division of Infectious Diseases. Patients were classified into the IDST group when joint consultation was approved and active IDST co-management was initiated. Therefore, patients who received active pharmacist-coordinated IDST co-management were classified into the IDST group. Patients were classified into the non-IDST (n-IDST) group when active IDST co-management was not initiated, regardless of usual antimicrobial stewardship support. In the n-IDST group, patients were managed by the attending physicians under usual care; however, pharmacist-led AST support, particularly anti-MRSA TDM and dose optimization, was provided when required. Patients directly managed by ID specialists without involvement of an IDST pharmacist remained in the n-IDST group because they did not receive the study intervention.

After approval, the IDST continued co-management until the patient’s condition stabilized or a treatment plan was established. The pharmacist-coordinated activities included triage of positive blood cultures, case summarization, participation in bedside rounds, focused physical assessment, documentation in the electronic medical record, antimicrobial dose optimization, and feedback or treatment recommendations to the attending physician.

The responsibilities of physicians and nurses were complementary. Non-specialist IDST physicians participated in daily bedside co-rounding and supported diagnostic evaluation and treatment or procedure planning with the pharmacist. ID specialists mainly supervised treatment strategies, supported the adjudication of complicated bacteraemia and metastatic foci, and provided guidance through comprehensive conferences held approximately once weekly. The ID nurse supported coordination and infection-control–related activities. In this programme, task shifting referred to the transfer of operational responsibilities including screening of positive blood cultures, chart review and case summarization, bedside assessment, bundle monitoring, documentation, and coordination of recommendations – from ID specialists to pharmacists. Diagnostic and therapeutic authority was not transferred and remained with the attending physicians, with ID specialist oversight for complex cases.

This redistribution of operational responsibilities was intended to allow limited ID specialist time to be focused on supervision and complex clinical decision-making; workload reduction was not directly evaluated in this study.

### Data collection

From the electronic medical records, we extracted data on age, sex, 28- and 90-day mortality, IDST intervention status, comorbidities, vital signs at blood culture collection (level of consciousness, blood pressure, respiratory rate, body temperature, intubation or mechanical ventilation, and cardiac arrest), type of anti-MRSA agent and concomitant drugs, and VCM minimum inhibitory concentration (MIC) for MRSA. The Charlson Comorbidity Index (CCI), Pitt Bacteraemia Score (PBS), and quick Sequential Organ Failure (qSOFA) score were calculated. We also recorded the presence of complicated bacteraemia or secondary infection (e.g. IE or abscess), computed tomography/magnetic resonance imaging (MRI) performance, and the achievement of each SAB bundle component.

### Definitions

The SAB bundle comprised QI of 12 items based on a study by Fukushima *et al.*, with minor modifications [18] (Table I). Achievement of each QI was determined based on actual implementation or completion documented in the medical record, not on whether the IDST recommendation was accepted. Each item counted as one point; the total score and adherence rate (≥75% defined as ≥9 points) were evaluated [19]. Language used in prior studies – e.g. ‘Antibiotic treatment therapy for patients with SAB should be adjusted according to renal function, and vancomycin monitoring should be performed’ – was modified and incorporated into QI 10 from the standpoint of appropriate antimicrobial use. Transoesophageal echocardiography (TEE) was not performed routinely in all patients with SAB. At our institution, TEE was proposed when IE remained clinically suspected after transthoracic echocardiogram (TTE), particularly in patients with relevant cardiac risk factors or persistent positive blood cultures. TEE was performed after discussion with, and approval by, the attending physician. Complicated bacteraemia was defined as MRSA bacteraemia with persistent bloodstream infection and/or secondary lesions, such as IE, device-related infections, osteomyelitis/pyogenic spondylitis, abscess formation, or other metastatic foci.

**Table I:** Quality indicators (QIs) for MRSA bacteraemia management: detailed list

QI categories
Blood culture
QI 1. Follow-up blood cultures after initiation of antimicrobial therapy
QI 2. Collection of repeat blood cultures should be performed until first negative blood culture
Echocardiography
QI 3. Transthoracic echocardiography should be performed in patients with diagnosed complicated SAB
QI 4. Transoesophageal echocardiography should be performed in patients with diagnosed complicated SAB
Source control
QI 5. After detection of SAB, eradicable focus should be removed
Antibiotics treatment
QI 6. Initial antibiotic therapy should be administered intravenously in patients with SAB
QI 7. Initial therapy should be intravenous anti-MRSA drug in patients with SAB
QI 8. Antibiotic therapy should be initiated within 24 h after first positive blood culture
QI 9. Appropriate duration of intravenous antibiotic treatment should be at least 14 days for uncomplicated SAB or at least 28 days for complicated SAB
QI 10. Appropriate dose and dosing interval of antimicrobial (VCM; AUC 400–600 μg·h/mL, TEIC; trough 20–40 μg/mL or DAP; ≥6 mg/kg)
QI 11. Intravenous-to-oral switch should not be performed in < 72 h
Other management
QI 12. Infectious disease specialist or IDST consultation should be performed in patients with SAB

MRSA, methicillin-resistant *Staphylococcus aureus*; SAB, *Staphylococcus aureus* bloodstream infection; VCM, vancomycin; AUC, blood concentration–time curve; TEIC, teicoplanin; DAP, daptomycin; IDST, Infectious Disease Support Team;;.

VCM and TEIC were defined as effective cases in this study if the blood concentrations remained within the range of AUC 400–600 μg·h/mL and trough 20–40 μg/mL, based on efficacy and safety criteria. For QI12 assessment, however, either ID specialist consultation or IDST consultation was counted as achievement.

### Outcomes

The primary outcome was SAB bundle adherence with and without IDST intervention. Secondary outcomes were 28-day mortality, and the factors associated with mortality were analysed. And 90-day mortality was assessed as an additional exploratory endpoint to evaluate intermediate-term outcomes. Additional analyses included comparisons by sex and between the pre-IDST and IDST periods. As a sensitivity analysis, QI implementation was also compared after excluding non-IDST patients directly managed by ID specialists.

### Statistical analysis

All analyses were performed using IBM SPSS Statistics version 26 (IBM Japan, Tokyo, Japan). For univariate analyses, categorical variables were compared using the χ^2^or Fisher’s exact test, and continuous variables were compared using the Mann–Whitney *U* test. Twenty-eight- and 90-day mortality rates were estimated using the Kaplan–Meier method and compared using log-rank tests. For risk factor evaluation, Cox proportional-hazards models were used to estimate hazard ratios (HRs), incorporating variables with *P* < 0.1 together with the CCI. Multi-collinearity was assessed using the variance inflation factor. Two-sided *P* < 0.05 was considered statistically significant.

## Results

### Cases

There were 101 cases in which MRSA was detected in blood cultures; of these, five patients died within 48 h after confirmation of blood culture positivity, no patients received VCM or TEIC without TDM, and 11 patients were excluded because population-based AUC prediction was not feasible, including one neonate/neonatal intensive care unit patient and 10 patients undergoing renal replacement therapy. A final total of 85 cases was included in the analysis. The median interval from initial positive blood culture to IDST intervention was one day. Eighty-five patients were included; the median age was 79 years, and 39 (45.9%) were female (Table II). The mean CCI, PBS, and qSOFA scores were 3.62, 1.53, and 0.80, respectively. Twenty-six patients (30.6%) died within 28 days, and 42 (49.4%) died within 90 days. IDST was performed in 39 patients (45.9%). The most common VCM MIC was 1 μg/mL in 71 cases (83.5%). Complicated bacteraemia occurred in 38 patients (44.7%), and secondary infections occurred in 31 patients (36.5%).

**Table II:** Patient characteristics between April 2020 and June 2025

Characteristics	Case and value
Case	85
Age, years, *median (IQR)*	79 (75–84)
Female, *N (%)*	39 (45.9)
Charlson Comorbidity Index score, *average ± SD*	3.62 ± 2.89
Pitt Bacteraemia score, *average ± SD*	1.53 ± 1.88
qSOFA, *average ± SD*	0.80 ± 0.89
IDST intervention should be performed in patients with SAB, *N (%)*	39 (45.9)
Time to IDST intervention, day, *median (IQR)*	1 (1,3)
Co-antimicrobial agent, *N (%)*	
Piperacillin/tazobactam	17 (20.0)
Meropenem	12 (14.1)
Ceftriaxone	10 (11.8)
Ampicillin/sulbactam	7 (8.2)
Cefepime	4 (4.7)
Cefmetazole	2 (2.4)
Fluconazole	2 (2.4)
Others	6 (7.1)
MRSA; Vancomycin MIC value, *N (%)*	
MIC=0.5 μg/mL	6 (7.1)
MIC=1 μg/mL	71 (83.5)
MIC=2 μg/mL	8 (9.4)
Complicated SAB, *N (%)*	38 (44.7)
Disseminated lesions (IE, pyogenic spondylitis, iliopsoas abscess, etc.), *N (%)*	31 (36.5)
Conducted CT or MRI of the torso, *N (%)*	64 (75.3)
Blood culture, *N (%)*	
QI 1. Follow-up blood cultures after initiation of antimicrobial therapy	79 (92.9)
QI 2. Collection of repeat blood cultures should be performed until first negative blood culture	69 (81.2)
Echocardiography, *N (%)*	54 (63.5)
QI 3. Transthoracic echocardiography should be performed in patients with diagnosed complicated SAB	54 (63.5)
QI 4. Transoesophageal echocardiography should be performed in patients with diagnosed complicated SAB	3 (3.5)
Source control, *N (%)*	
QI 5. After detection of SAB, eradicable focus should be removed	51 (60.0)
Removal of intravascular catheter	39 (45.9)
Surgical intervention/drainage	16 (18.9)
Antibiotics treatment, *N (%)*	
QI 6. Initial antibiotic therapy should be administered intravenously in patients with SAB	85 (100)
QI 7. Initial therapy should be intravenous anti-MRSA drug in patients with SAB	71 (83.5)
QI 8. Antibiotic therapy should be initiated within 24 h after first positive blood culture	46 (54.1)
QI 9. Appropriate duration of intravenous antibiotic treatment should be at least 14 days for uncomplicated SAB or at least 28 days for complicated SAB	51 (60.0)
QI 10. Appropriate dose and dosing interval of antimicrobial (VCM; AUC 400–600 μg·h/mL, TEIC; trough 20–40 μg/mL or DAP; ≥6 mg/kg)	71 (83.5)
VCM use	82 (96.5)
AUC 400–600 μg·h/mL attainment per day within 2 days of start	69 (84.1)
TEIC use	1 (1.2)
Treatment starts at VCM, switched from day 2	1 (1.2)
Trough 20–40 μg/mL attainment within 2 days of start	1 (100)
DAP use	5 (5.9)
Treatment starts at VCM, switched from day 2	2 (2.4)
Dose ≥6 mg/kg	5 (100)
QI 11. Intravenous-to-oral switch should not be performed in <72 h	85 (100)
QI 12. Infectious disease specialist or IDST consultation should be performed in patients with SAB	56 (65.9)
Bundle	
Total score, *median (IQR)*	9 (7, 10)
Bundle attainment ≥ 75%, *N (%)*	49 (57.6)

IQR, interquartile range; SD, standard deviation; qSOFA, quick Sequential Organ Failure; IDST, Infectious Disease Support Team; SAB, *Staphylococcus aureus* bloodstream infection; MIC, minimum inhibitory concentration; IE, infective endocarditis; CT, computed tomography; MRI, magnetic resonance imaging; QI, quality indicator; MRSA, methicillin-resistant *Staphylococcus aureus*; VCM, vancomycin; AUC, area under the blood concentration–time curve; TEIC, teicoplanin; DAP, daptomycin.; ;;;;;.

Data for each characteristic were presented as frequency (proportion) and median or average values.

IDST co-management was initiated in 39 of 85 patients (45.9%). Patients without active IDST co-management included those already directly managed by ID specialists, those for whom the primary team declined joint consultation because they considered usual care sufficient, and those for whom active treatment escalation was not desired. Even in these cases, pharmacist-coordinated support for appropriate antimicrobial use, mainly TDM, was provided when required.

### SAB bundle adherence and IDST intervention

Overall bundle adherence ≥75% was achieved in 57.6% of cases. Item-level performance was as follows (Table II): follow-up blood cultures in 79 cases (92.9%), confirmation of negative conversion in 69 (81.2%), echocardiography in 54 (63.5%), including TEE in three, source control in 51 (60.0%), catheter removal in 39 (45.9%), drainage/surgery in 16 (18.9%), and initiation of antimicrobial therapy within 24 h in 46 (54.1%). Appropriate dosing and administration were observed in 71 patients (83.5%), and the recommended treatment duration was completed in 51 (60.0%) patients. With IDST intervention, bundle adherence ≥75% using QI1–12 was significantly higher than without IDST intervention (79.5% vs 39.1%, *P* < 0.001, Table III). In a sensitivity analysis excluding QI12, the total bundle score remained significantly higher in the IDST group (8 vs 8 points, *P*=0.003), while bundle adherence ≥75% was numerically higher but did not reach statistical significance (46.2% vs 26.1%, *P*=0.070). The IDST group had significantly higher echocardiography rates (89.7% vs 41.3%, *P* < 0.001) and completion of the recommended duration (76.9% vs 45.7%, *P*=0.004). The total bundle score was higher with intervention (10 vs 8 points, *P* < 0.001). The intervention group also tended to have more complicated bacteraemia (56.4% vs 34.8%, *P*=0.075) and secondary infections (46.2% vs 28.3%, *P*=0.138).

**Table III:** Comparison adherence to treatment bundles between intervention with (IDST) and without (n-IDST)

Characteristics	IDST	n-IDST	*P*-value
Case	39	46	
Age, years, *median (IQR)*	79 (74, 84)	81 (75, 84)	0.377
Female, *N (%)*	18 (46.2)	21 (45.7)	1.000
Charlson Comorbidity Index score, *average ± SD*	4.21 ± 3.20	3.13 ± 2.49	0.153
Pitt Bacteraemia score, *average ± SD*	1.51 ± 1.95	1.54 ± 1.81	0.720
qSOFA, *average ± SD*	0.74 ± 0.95	0.85 ± 0.83	0.367
28-day mortality, *N (%)*	7 (17.9)	19 (41.3)	0.033
90-day mortality, *N (%)*	14 (35.9)	28 (60.9)	0.038
Complicated SAB, *N (%)*	22 (56.4)	16 (34.8)	0.075
Disseminated lesions (IE, pyogenic spondylitis, iliopsoas abscess, etc.), *N (%)*	18 (46.2)	13 (28.3)	0.138
Bundle QI 1–12, *N (%)*			
Blood culture			
QI 1. Follow-up blood cultures after initiation of antimicrobial therapy	37 (94.9)	42 (91.3)	0.683
QI 2. Collection of repeat blood cultures should be performed until first negative blood culture	34 (87.2)	35 (76.1)	0.267
Echocardiography			
QI 3. Transthoracic echocardiography should be performed in patients with diagnosed complicated SAB	35 (89.7)	19 (41.3)	<0.001
QI 4. Transoesophageal echocardiography should be performed in patients with diagnosed complicated SAB	3 (7.7)	0 (0)	0.093
Source control			
QI 5. After detection of SAB, eradicable focus should be removed	26 (66.7)	25 (54.3)	0.351
Antibiotics treatment			
QI 6. Initial antibiotic therapy should be administered intravenously in patients with SAB	39 (100)	46 (100)	1.000
QI 7. Initial therapy should be intravenous anti-MRSA drug in patients with SAB	31 (79.5)	40 (87.0)	0.392
QI 8. Antibiotic therapy should be initiated within 24 h after first positive blood culture	21 (53.8)	25 (54.3)	1.000
QI 9. Appropriate duration of intravenous antibiotic treatment should be at least 14 days for uncomplicated SAB or at least 28 days for complicated SAB	30 (76.9)	21 (45.7)	0.004
QI 10. Appropriate dose and dosing interval of antimicrobial (VCM; AUC 400–600 μg·h/mL, TEIC; trough 20–40 μg/mL or DAP; ≥6 mg/kg)	31 (79.5)	40 (87.0)	0.392
QI 11. Intravenous-to-oral switch should not be performed in < 72 h	39 (100)	46 (100)	1.000
Other management			
QI 12. Infectious disease specialist or IDST consultation should be performed in patients with SAB	39 (100)	17 (37.0)	<0.001
Bundle total score QI 1–11, *median (IQR)*	8 (8, 10)	8 (6, 9)	0.003
Bundle attainment ≥ 75%, *N (%)*	18 (46.2)	12 (26.1)	0.070
Bundle total score QI 1–12, *median (IQR)*	10 (9, 11)	8 (7, 9)	<0.001
Bundle attainment ≥ 75%, *N (%)*	31 (79.5)	18 (39.1)	<0.001

IDST, Infectious Disease Support Team; n-IDST, non-IDST; IQR, interquartile range; SD, standard deviation; qSOFA, quick Sequential Organ Failure; SAB, *Staphylococcus aureus* bloodstream infection; IE, infective endocarditis; MRSA, methicillin-resistant *Staphylococcus aureus*; VCM, vancomycin; AUC, area under the blood concentration–time curve; TEIC, teicoplanin; DAP, daptomycin.

Data for each characteristic were presented as frequency (proportion) and median or average values.

Categorical variables were analysed using the χ^2^-square test or Fisher’s exact test, and continuous variables were analysed using the Mann–Whitney *U* test. Statistical significance was defined as *P* < 0.05.

In an additional period-based analysis comparing the pre-IDST and IDST periods, the total bundle score was significantly higher during the IDST period than during the pre-IDST period (9 [8,10] vs 8 [7,9], *P*=0.007). Bundle attainment ≥75% was numerically higher during the IDST period, although the difference did not reach statistical significance (64.9% vs 42.9%, *P*=0.065) (Supplementary Table II).

In a sensitivity analysis excluding n-IDST patients directly managed by ID specialists, TTE performance (89.7% vs 24.1%, *P* < 0.001) and completion of the recommended treatment duration (76.9% vs 48.3%, *P*=0.021) remained significantly higher in the IDST group (Supplementary Table III).

### Factors associated with mortality

Compared with the non-intervention (n-IDST) group, the IDST group had lower 28-day mortality (17.9% vs 41.3%, *P*=0.033) and 90-day mortality (35.9% vs 60.9%, *P*=0.038) (Table III). In the univariate analysis, the 28-day mortality group had a higher proportion of females (*P*=0.020) and higher PBS (*P*=0.005) and qSOFA (*P* < 0.001) scores than the survival group (Table IV). Lower mortality was associated with the following factors: performance of follow-up blood cultures (*P* < 0.001), confirmation of negative conversion (*P* < 0.001), echocardiography (*P*=0.003), source-control measures (*P*=0.014), completion of the recommended duration (*P* < 0.001), IDST intervention (*P*=0.033), and higher bundle score (*P* < 0.001).

**Table IV:** Comparison of patient characteristics by 28-day and 90-day mortality status

Characteristics	28-day mortality			90-day mortality		
	+	−	*P*-value	+	−	*P*-value
Case	26	59		42	43	
Age, years, *median (IQR)*	82 (76, 84)	78 (73, 84)	0.111	82 (76, 85)	75 (73, 82)	0.007
Female, *N (%)*	17 (65.4)	22 (37.3)	0.020	20 (47.6)	19 (44.2)	0.920
Charlson Comorbidity Index score, *average ± SD*	4.23 ± 3.08	3.36 ± 2.76	0.230	4.29 ± 2.95	2.98 ± 2.67	0.029
Pitt Bacteraemia score, *average ± SD*	2.23 ± 1.89	1.22 ± 1.79	0.005	1.83 ± 1.77	1.23 ± 1.93	0.032
qSOFA, *average ± SD*	1.38 ± 0.92	0.54 ± 0.74	<0.001	1.12 ± 0.91	0.49 ± 0.76	<0.001
Complicated SAB, *N (%)*	12 (46.2)	26 (44.1)	1.000	17 (40.5)	21 (48.8)	0.515
Disseminated lesions (IE, pyogenic spondylitis, iliopsoas abscess, etc.), *N (%)*	10 (38.5)	21 (35.6)	0.993	14 (33.3)	17 (39.5)	0.654
Bundle QI 1–12, *N (%)*						
Blood culture						
QI 1. Follow-up blood cultures after initiation of antimicrobial therapy	20 (76.9)	59 (100)	<0.001	36 (85.7)	43 (100)	0.012
QI 2. Collection of repeat blood cultures should be performed until first negative blood culture	12 (46.2)	57 (96.6)	<0.001	26 (61.9)	43 (100)	<0.001
Echocardiography						
QI 3. Transthoracic echocardiography should be performed in patients with diagnosed complicated SAB	10 (38.5)	44 (74.6)	0.003	21 (50.0)	33 (76.7)	0.020
QI 4. Transoesophageal echocardiography should be performed in patients with diagnosed complicated SAB	0 (0)	3 (5.1)	0.550	1 (2.4)	2 (4.7)	1.000
Source control						
QI 5. After detection of SAB, eradicable focus should be removed	10 (38.5)	41 (69.5)	0.014	21 (50.0)	30 (69.8)	0.101
Antibiotics treatment						
QI 6. Initial antibiotic therapy should be administered intravenously in patients with SAB	26 (100)	59 (100)	1.000	42 (100)	43 (100)	1.000
QI 7. Initial therapy should be intravenous anti-MRSA drug in patients with SAB	22 (84.6)	49 (83.1)	1.000	36 (85.7)	35 (81.4)	0.771
QI 8. Antibiotic therapy should be initiated within 24 h after first positive blood culture	14 (53.8)	32 (54.2)	1.000	22 (52.4)	24 (55.8)	0.920
QI 9. Appropriate duration of intravenous antibiotic treatment should be at least 14 days for uncomplicated SAB or at least 28 days for complicated SAB	1 (3.8)	50 (84.7)	<0.001	13 (31.0)	38 (88.4)	<0.001
QI 10. Appropriate dose and dosing interval of antimicrobial (VCM; AUC 400–600 μg·h/mL, TEIC; trough 20–40 μg/mL or DAP; ≥6 mg/kg)	22 (84.6)	49 (83.1)	1.000	36 (85.7)	35 (81.4)	0.771
QI 11. Intravenous-to-oral switch should not be performed in < 72 h	26 (100)	59 (100)	1.000	42 (100)	43 (100)	1.000
Other management						
QI 12. Infectious disease specialist or IDST consultation should be performed in patients with SAB	12 (46.2)	44 (74.6)	0.014	24 (57.1)	32 (74.4)	0.147
Bundle total score, *median (IQR)*	7 (6, 8)	9 (9, 10)	<0.001	8 (6, 9)	9 (9, 10)	<0.001
Bundle attainment ≥ 75%, *N (%)*	4 (15.4)	45 (76.3)	<0.001	16 (38.1)	33 (76.7)	<0.001

IQR, interquartile range; SD, standard deviation; qSOFA, quick Sequential Organ Failure; IE, infective endocarditis; SAB, *Staphylococcus aureus* bloodstream infection; MRSA, methicillin-resistant *Staphylococcus aureus*; VCM, vancomycin; AUC, area under the blood concentration–time curve; TEIC, teicoplanin; DAP, daptomycin; IDST, Infectious Disease Support Team.

Data for each characteristic were presented as frequency (proportion) and median or average values.

Categorical variables were analysed using the χ^2^ test or Fisher’s exact test, and continuous variables were analysed using the Mann–Whitney *U* test. Statistical significance was defined as *P* < 0.05.

The 90-day mortality group was older (*P*=0.007) and had higher CCI (*P*=0.029), PBS (*P*=0.032), and qSOFA (*P* < 0.001) scores. Lower mortality was associated with the following factors: performance of follow-up blood cultures (*P*=0.012), confirmation of negative conversion (*P* < 0.001), echocardiography (*P*=0.020), completion of the recommended duration (*P* < 0.001), IDST intervention (*P*=0.038), and higher bundle score (*P* < 0.001).

### Survival curves and Cox proportional-hazards analysis

**Figure 2:**
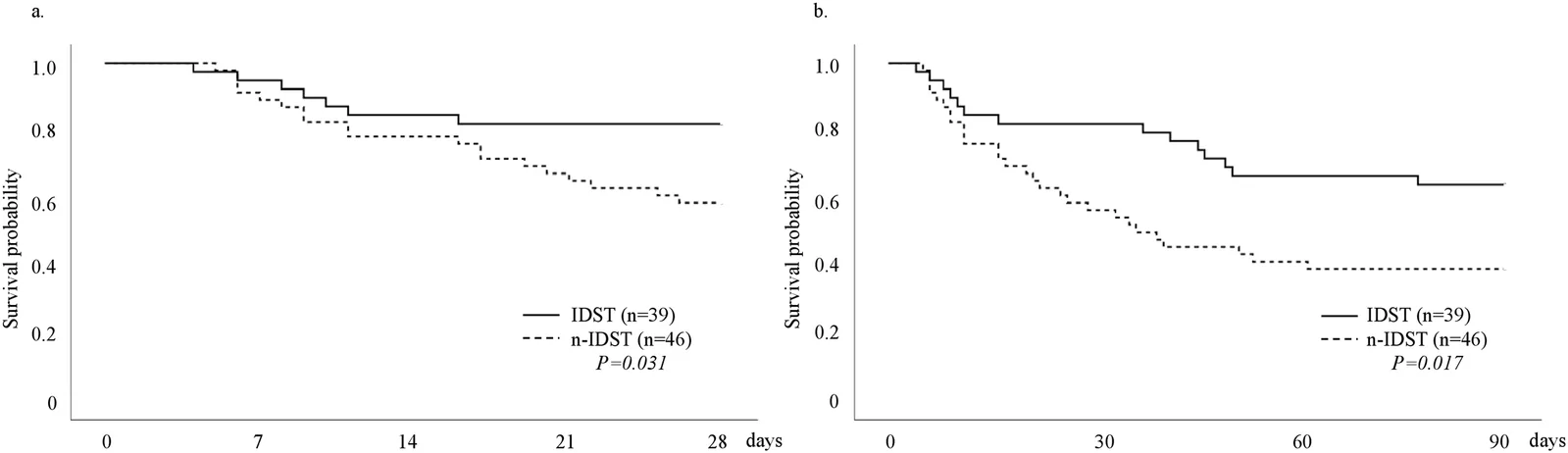
Kaplan–Meier survival curves for mortality between intervention with (IDST) and without (n-IDST). a. 28-day mortality. b. 90-day mortality. A log-rank test was performed to assess the significance of the differences between survival curves. Statistical significance was defined as *P* < 0.05. IDST, infectious disease support team; n-IDST, non-IDST

In the Kaplan–Meier analysis, the IDST group had significantly lower 28-day (log-rank *P*=0.031) and 90-day mortality (*P*=0.017) than the n-IDST group (Figure 2). In the Cox model, the independent factors for 28-day mortality were female sex (HR 2.558, 95% confidence interval [CI]: 1.124–5.818), PBS (HR 1.234, 95% CI: 1.038–1.466), and IDST (HR 0.328, 95% CI: 0.136–0.792) (Table V). Independent factors for 90-day mortality were age (HR 1.036, 95% CI: 1.001–1.073), CCI (HR 1.133, 95% CI: 1.018–1.261), PBS (HR 1.236, 95% CI: 1.068–1.430), and IDST intervention (HR 0.427, 95% CI: 0.215–0.846). The VIF fluctuated within the range of 1.076–1.195.

**Table V:** Cox proportional-hazards model of 28-day and 90-day mortality

	28-day mortality			90-day mortality		
	HR	95 % CI	*P*-value	HR	95 % CI	*P*-value
Age				1.036	1.001–1.073	0.046
Female	2.558	1.124–5.818	0.025			
Charlson Comorbidity Index score	1.131	0.993–1.287	0.063	1.133	1.018–1.261	0.022
Pitt Bacteraemia score	1.234	1.038–1.466	0.017	1.236	1.068–1.430	0.005
IDST intervention	0.328	0.136–0.792	0.013	0.427	0.215–0.846	0.015

HR, hazard ratio; CI, confidence interval; IDST, Infectious Disease Support Team.

Statistical significance was defined as *P* < 0.05. All tests were two-sided, and 95% confidence intervals were calculated.

## Discussion

In this study, pharmacist-coordinated IDST co-management for MRSA bacteraemia was associated with higher SAB bundle adherence and lower 28- and 90-day mortality. Although qSOFA and PBS scores were similar between the groups, the intervention group did not appear to represent a clearly ‘easier’ case mix, as complicated bacteraemia and secondary foci were numerically more frequent in this group. However, this interpretation should be cautious because the diagnostic workup for IE and metastatic foci may not have been complete in all patients, particularly in those without active IDST co-management. The main difference from the previous pharmacist-led AST framework was not antimicrobial optimization alone, but the integration of antimicrobial stewardship with structured bedside assessment, diagnostic support, source-control planning, and treatment-duration review. These findings suggest that a multi-disciplinary IDST model may improve the reliability of evidence-based SAB care processes in settings with limited ID physician resources.

In the multi-variate analysis, IDST participation was independently associated with lower 28- and 90-day mortality rates. Building on this finding, we examined why pharmacist-coordinated IDST might improve patient outcomes. First, the bundle adherence rate ≥75% was approximately 40% higher in the IDST group than in the n-IDST group. Because QI12 may partially overlap with the IDST intervention, we also evaluated bundle adherence after excluding QI12. In this sensitivity analysis, the bundle adherence rate ≥75% was remained approximately 20% higher even in an analysis excluding QI12, but the difference in ≥75% bundle attainment was attenuated and did not reach statistical significance. In the additional period-based analysis, the total bundle score was significantly higher during the IDST period than during the pre-IDST period (9 [8,10] vs 8 [7,9], *P*=0.007), although bundle attainment ≥75% was numerically higher but did not reach statistical significance (64.9% vs 42.9%, *P*=0.065). Therefore, we interpreted the IDST as a co-management model that may improve the reliability of bundle-based care, while acknowledging that the period-based comparison is susceptible to temporal confounding. In IDST vs n-IDST, the most significant contributors to this gap were echocardiographic performance and completion of the recommended treatment duration, which differed by approximately 48% and 31%, respectively. Moreover, the IDST group also numerically higher underwent follow-up blood culture (+3.6%), confirmation of blood culture clearance (+11.1%), and source control (+12.4%). Previous studies on SAB have reported that delivering these steps as an integrated care process, including echocardiography, early intravenous antimicrobial therapy, and timely source-control measures, is associated with lower 30-day mortality [15,20]. In contrast, Willekens *et al.* [19] found no consistent association between single QIs and mortality. Our results are concordant with this view; rather than any single QI driving survival, the accumulation of multiple adhered QIs appears to underlie the observed outcome benefit. Consistently, Nagao *et al.* reported that adherence to ≥4 of 5 QIs was associated with reduced mortality [21], a finding reinforced by the present study. Thus, IDST activity should be understood not as a single intervention but as a programme that comprehensively promotes a sequence of processes – timely diagnostics, appropriate source control, and disciplined management of treatment duration – to reliably translate recommended care into clinical practice. Importantly, the bundle served as a structured framework for IDST co-management, not as a simple checklist. Implementation of SAB care processes requires bedside assessment, evaluation of symptoms and physical findings, identification of possible metastatic foci, and coordination of diagnostic testing and source control. Thus, the observed improvement should be interpreted as the effect of team-based ID support built around the bundle, rather than the effect of checklist use alone.

Moreover, pharmacist-coordinated task shifting is not merely a strategy to reduce physicians’ workload; it also confers process advantages to clinical care. Pharmacists acted as workflow coordinators by triaging patients with positive blood cultures, conducting targeted chart reviews, summarizing clinical problems, participating in bedside rounds, documenting assessments, and providing concrete recommendations to the attending physicians. Taken together, these activities narrow the time gap between microbiological information and bedside decision-making, thereby reducing delays in appropriate therapy initiation and source control [22,23]. In contrast, physicians retained responsibility for diagnostic judgement and complex therapeutic decisions. Non-specialist IDST physicians collaborated with pharmacists during daily bedside assessment, whereas ID specialists provided oversight of treatment strategies and supported the adjudication of complicated bacteraemia and metastatic foci through regular conferences. This division of labour may explain how the pharmacist-coordinated IDST promoted bundle-based care despite limited ID physician resources – may help ensure timely actions and appropriate antimicrobial use, preventing omissions or deviations at key inpatient workflow steps. This division of labour is consistent with previous reports showing that co-management by ID pharmacists and physicians strengthens care and improves processes [24].

As a complement to these pharmacist-coordinated efforts, our findings suggest that physicians can focus on diagnostic strategies and complex decision-making processes, while pharmacists support the reliability of routine bundle implementation. However, our results should not be interpreted as showing that this model can be implemented without any ID physician involvement. Rather, the findings support a task-shifted co-management model in which pharmacists coordinate bundle-based care under intermittent ID specialist oversight, such as regular case conferences or teleconsultation. These elements can be implemented even in hospitals with limited access to ID specialists, provided there is an environment that allows for consultation with experts; they can be strengthened through telemedicine consultations and regular case conferences, which is consistent with and supports existing reports [25]. On the other hand, this approach may require further evaluation regarding its applicability to hospitals that lack access to ID expertise.

In contrast, the finding that female sex was an independent risk factor for 28-day mortality in patients with SAB was unexpected. Although male mortality is generally higher in IDs [[26], [27], [28], [29]], our results were contrasting. However, recent data comparing sex-related differences in SAB mortality rates have reported significantly higher rates among female patients [30,31]. Notably, a meta-analysis by Westgeest *et al.* indicated 18% higher odds of SAB mortality in female patients than in males [31]. These reports discuss sex-related differences in the time from the onset of bacteraemia symptoms to seeking medical care. We similarly considered differences in time to presentation, symptom perception, help-seeking, comorbidity patterns, and unmeasured confounding factors. In our study, female tended to present with more physiological abnormalities (higher PBS and qSOFA scores) (Supplementary Table I). These findings suggest later recognition of more advanced disease by the time blood cultures were drawn. These findings highlight the challenges of detecting SAB in patients of either sex as quickly as possible and avoiding delays from initial abnormal vital signs to definitive treatment.

This study had some limitations. This study employed a retrospective, single-centre design with a limited number of cases, and selection and information biases were present. Because IDST co-management required approval from the attending physician, group allocation was not random and may have been influenced by clinical judgement, including whether the primary team considered usual care sufficient or whether escalation of active treatment was appropriate. Therefore, residual selection bias related to the decision to initiate IDST co-management cannot be excluded. Furthermore, the inclusion of cases that were directly managed by ID specialists in the non-intervention group remains a source of selection bias. Moreover, when the IDST intervenes after a positive blood culture, immortal-time bias is a theoretical concern. We acknowledge that while excluding early deaths in this study mitigates this bias, it does not eliminate it. In non-intervention patients with inadequate examination and diagnosis, there was a high likelihood of misclassification of complex SAB and metastatic lesions, which may have introduced a bias affecting the outcomes. In fact, the infrequent use of TEE may have led to missed diagnoses of IE. Due to sample size limitations, subgroup analyses (e.g. MIC, device presence, and cause of bacteraemia) were not possible. In addition, we did not measure intermediate-term outcomes, such as time-to-appropriate therapy, time-to-source control, length of stay, re-admissions, nephrotoxicity, or cost-key implementation endpoints. Finally, we were unable to fully adjust for confounding factors such as underlying conditions regarding 90-day mortality. MRSA bacteraemia frequently occurs in immunocompromised patients, patients with cancer, and patients with long-term catheter placement due to prolonged hospitalization [[32], [33], [34], [35], [36], [37]], making it impossible to rule out the impact of mortality from these underlying conditions. Therefore, the 90-day mortality analysis should be interpreted as supportive and exploratory.

A pharmacist-coordinated IDST can implement SAB bundles at the bedside, potentially translating process reliability into clinically meaningful survival gains. In settings with limited physician ID availability, task-shifted, protocolled co-management offers a feasible path for higher-quality care. Strengthening IDST interventions by incorporating bundles, systematizing interventions, collaborating with other departments, and supporting appropriate antimicrobial use can lead to a more robust treatment system.

## CRediT authorship contribution statement

**Aiju Endo:** Writing – original draft, Visualization, Validation, Software, Project administration, Methodology, Investigation, Formal analysis, Data curation, Conceptualization. **Yuki Hanai:** Writing – review & editing, Validation, Supervision, Project administration, Methodology, Conceptualization. **Takahiro Mikawa:** Writing – review & editing, Validation, Supervision, Methodology, Formal analysis, Conceptualization. **Daiki Asakawa:** Writing – review & editing, Investigation, Data curation. **Taiki Ishibe:** Writing – review & editing, Investigation, Data curation. **Yu Nakane:** Writing – review & editing, Investigation, Data curation. **Misako Tanaka:** Writing – review & editing, Investigation, Data curation, Conceptualization. **Daichi Mitsui:** Writing – review & editing, Investigation, Data curation, Conceptualization. **Shinji Kido:** Writing – review & editing, Investigation, Data curation, Conceptualization. **Takeshi Nonaka:** Writing – review & editing, Investigation, Data curation, Conceptualization. **Ken Fujimori:** Writing – review & editing, Investigation, Data curation, Conceptualization. **Nozomi Kudo:** Writing – review & editing, Investigation, Data curation, Conceptualization. **Yuta Nakadoi:** Writing – review & editing, Investigation, Data curation, Conceptualization. **Yukimasa Kobayashi:** Writing – review & editing, Investigation, Data curation, Conceptualization. **Rintaro Negishi:** Writing – review & editing, Investigation, Data curation, Conceptualization. **Ayaka Fujimori:** Writing – review & editing, Investigation, Data curation, Conceptualization. **Mika Takatori:** Writing – review & editing, Investigation, Data curation, Conceptualization. **Yasuyuki Natsume:** Writing – review & editing, Investigation, Data curation, Conceptualization. **Kaori Matsumoto:** Writing – review & editing, Supervision, Project administration. **Kazuaki Matsumoto:** Writing – review & editing, Supervision, Project administration, Methodology, Conceptualization.

## Consent for publication

This study does not include individual data (images or identifiable details).

## Data availability

The datasets generated and/or analysed in the current study are available from the corresponding author upon reasonable request.

## Ethics statement

This retrospective study was approved by the Clinical and Genomic Research Ethics Review Committee (Approval No. 2023-70), and the requirement for written informed consent was waived because of the retrospective design. An opt-out notice was posted on the hospital’s website. The study adhered to the Declaration of Helsinki and the applicable regulations.

## Declaration of generative AI and AI-assisted technologies in the manuscript preparation process

During the preparation of this work, the authors used ChatGPT (OpenAI) for English-language translation and editing. After using this tool/service, the authors reviewed and edited the content as needed and take full responsibility for the content of the published article.

## Funding sources

None.

## Conflict of interest statement

The authors have no relevant financial or non-financial interests to disclose.
