# Supplementary material for: Pharmacist-coordinated IDST treatment bundle achievement and impact on mortality in MRSA bacteraemia: a single-centre retrospective cohort study
Source: Infect Prev Pract. 2026 Aug 8;8(4):100578. doi: 10.1016/j.infpip.2026.100578 (PMC13545305; doi:10.1016/j.infpip.2026.100578)
Supplement: Supplementary file 1 [file mmc1.docx]

**Supplementary information**

Supplementary Table I. Comparison between female and male

Data for each characteristic were presented as frequency (proportion) and median or average values.

Categorical variables were analyzed using the chi-square test or Fisher's exact test, and continuous variables were analyzed using the Mann–Whitney U test. Statistical significance was defined as p< 0.05.

CT, computed tomography; IDST, infectious disease support team; IE, infective endocarditis; IQR, interquartile range; MRI, magnetic resonance imaging; MRSA, methicillin-resistant *Staphylococcus aureus*; QI, quality indicator; qSOFA, quick Sequential Organ Failure; SAB, *Staphylococcus aureus* bloodstream infection; SD, standard deviation

| Characteristics | Female | Male | P- value |
| --- | --- | --- | --- |
| Case | 39 | 46 |  |
| Age, years, *median (IQR)* | 80 (74, 85) | 79 (75, 84) | 0.694 |
| Charlson Comorbidity Index score, *average ± SD* | 3.44 ± 2.97 | 3.78 ± 2.81 | 0.473 |
| Pitt Bacteremia score, *average ± SD* | 2.08 ± 1.89 | 1.07 ± 1.73 | <0.001 |
| qSOFA, *average ± SD* | 1.13 ± 0.99 | 0.52 ± 0.68 | 0.003 |
| Complicated SAB, *n (%)* | 19 (48.7) | 19 (41.3) | 0.641 |
| Disseminated lesions (IE, pyogenic spondylitis, iliopsoas abscess, etc.), *n (%)* | 15 (38.5) | 16 (34.8) | 0.901 |
| Bundle QI 1-12, *n (%)* |  |  |  |
| Blood culture |  |  |  |
| QI 1. Follow-up blood cultures after initiation of antimicrobial therapy | 35 (89.7) | 44 (95.7) | 0.406 |
| QI 2. Collection of repeat blood cultures should be performed until first negative blood culture | 28 (71.8) | 41 (89.1) | 0.053 |
| Echocardiography |  |  |  |
| QI 3. Transthoracic echocardiography should be performed in patients with diagnosed complicated SAB | 22 (56.4) | 32 (69.6) | 0.303 |
| QI 4. Transesophageal echocardiography should be performed in patients with diagnosed complicated SAB | 1 (2.6) | 2 (4.3) | 1.000 |
| Source control |  |  |  |
| QI 5. After detection of SAB, eradicable focus should be removed | 25 (64.1) | 26 (56.5) | 0.625 |
| Antibiotics treatment |  |  |  |
| QI 6. Initial antibiotic therapy should be administered intravenously in patients with SAB | 39 (100) | 46 (100) | 1.000 |
| QI 7. Initial therapy should be intravenous anti-MRSA drug in patients with SAB | 39 (100) | 46 (100) | 1.000 |
| QI 8. Antibiotic therapy should be initiated within 24 h after first positive blood culture | 24 (61.5) | 22 (47.8) | 0.296 |
| QI 9. Appropriate duration of intravenous antibiotic treatment should be at least 14 days for uncomplicated SAB, or at least 28 days for complicated SAB | 20 (51.3) | 31 (67.4) | 0.198 |
| QI 10. Appropriate dose and dosing interval of antimicrobial  (VCM; AUC ≥400, TEIC; trough ≥20 µg/mL or DAP; ≥6mg/kg) | 31 (79.5) | 40 (87.0) | 0.392 |
| QI 11. Intravenous-to-oral switch should not be performed in < 72 h | 39 (100) | 46 (100) | 1.000 |
| Other management |  |  |  |
| QI 12. Infectious disease specialist or IDST consultation should be performed in patients with SAB | 25 (64.1) | 31 (67.4) | 0.929 |
| Bundle total score, *median (IQR)* | 9 (7, 9) | 9 (8, 10) | 0.911 |
| Bundle attainment ≥ 75%, *n (%)* | 23 (59.0) | 26 (56.5) | 1.000 |

Supplementary Table Ⅱ. Comparison between IDST activities before and after in bundle adherence

Data for each characteristic are presented as frequency (proportion) and the median or average value.

Categorical variables were analyzed with the chi-square or Fisher's exact test, and continuous variables with the Mann–Whitney U test. Statistical significance was defined as p < 0.05.

DAP; daptomycin, IDST; infectious disease support team, IQR; interquartile range, MRSA; methicillin-resistant *Staphylococcus aureus*, QI; quality indicator, SAB; *Staphylococcus aureus* blood stream infection, SD; standard deviation, TEIC; teicoplanin, VCM; vancomycin

| Characteristics | Before | After | P- value |
| --- | --- | --- | --- |
| Case | 28 | 57 |  |
| Bundle QI 1-12, *n (%)* |  |  |  |
| Blood culture |  |  |  |
| QI 1. Follow-up blood cultures after initiation of antimicrobial therapy | 27 (96.4) | 52 (91.2) | 0.659 |
| QI 2. Collection of repeat blood cultures should be performed until first negative blood culture | 22 (78.6) | 47 (82.5) | 0.770 |
| Echocardiography |  |  |  |
| QI 3. Transthoracic echocardiography should be performed in patients with diagnosed complicated SAB | 10 (35.7) | 44 (77.2) | <0.001 |
| QI 4. Transesophageal echocardiography should be performed in patients with diagnosed complicated SAB | 0 (0) | 3 (5.3) | 0.548 |
| Source control |  |  |  |
| QI 5. After detection of SAB, eradicable focus should be removed | 17 (60.7) | 34 (59.6) | 1.000 |
| Antibiotics treatment |  |  |  |
| QI 6. Initial antibiotic therapy should be administered intravenously in patients with SAB | 28 (100) | 57 (100) | 1.000 |
| QI 7. Initial therapy should be intravenous anti-MRSA drug in patients with SAB | 24 (85.7) | 47 (82.5) | 1.000 |
| QI 8. Antibiotic therapy should be initiated within 24 hr after first positive blood culture | 18 (64.3) | 28 (49.1)) | 0.248 |
| QI 9. Appropriate duration of intravenous antibiotic treatment should be at least 14 days for uncomplicated SAB, or at least 28 days for complicated SAB | 15 (53.6) | 36 (63.2) | 0.482 |
| QI 10. Appropriate dose and dosing interval of antimicrobial  (VCM; AUC 400-600 μg·h/mL, TEIC; trough 20-40 µg/mL or DAP; ≥6mg/kg) | 24 (85.7) | 47 (82.5) | 1.000 |
| QI 11. Intravenous-to-oral switch should not be performed in < 72 hr | 28 (100) | 57 (100) | 1.000 |
| Other management |  |  |  |
| QI 12. Infectious disease specialist or IDST consultation should be performed in patients with SAB | 6 (21.4) | 50 (87.7) | <0.001 |
| Bundle total score, *median (IQR)* | 8 (7, 9) | 9 (8, 10) | 0.007 |
| Bundle attainment ≥ 75%, *n (%)* | 12 (42.9) | 37 (64.9) | 0.065 |

Supplementary Table 3. Sensitivity analysis of QI implementation after excluding non-IDST patients directly managed by infectious disease specialists

Categorical variables were analyzed with the chi-square or Fisher's exact test, and continuous variables with the Mann–Whitney U test. Statistical significance was defined as p < 0.05.

IDST; infectious disease support team, IQR; interquartile range, MRSA; methicillin-resistant Staphylococcus aureus, QI; quality indicator, SAB; Staphylococcus aureus blood stream infection

| Characteristics | IDST | n-IDST | P- value |
| --- | --- | --- | --- |
| Case | 39 | 29 |  |
| Bundle QI 1-11, *n (%)* |  |  |  |
| Blood culture |  |  |  |
| QI 1. Follow-up blood cultures after initiation of antimicrobial therapy | 37 (94.9) | 25 (86.2) | 0.390 |
| QI 2. Collection of repeat blood cultures should be performed until first negative blood culture | 34 (87.2) | 22 (75.9) | 0.336 |
| Echocardiography |  |  |  |
| QI 3. Transthoracic echocardiography should be performed in patients with diagnosed complicated SAB | 35 (89.7) | 7 (24.1) | <0.001 |
| QI 4. Transesophageal echocardiography should be performed in patients with diagnosed complicated SAB | 3 (7.7) | 0 (0) | 0.255 |
| Source control |  |  |  |
| QI 5. After detection of SAB, eradicable focus should be removed | 26 (66.7) | 17 (58.6) | 0.613 |
| Antibiotics treatment |  |  |  |
| QI 6. Initial antibiotic therapy should be administered intravenously in patients with SAB | 39 (100) | 29 (100) | 1.000 |
| QI 7. Initial therapy should be intravenous anti-MRSA drug in patients with SAB | 31 (79.5) | 26 (89.7) | 0.331 |
| QI 8. Antibiotic therapy should be initiated within 24 hr after first positive blood culture | 21 (53.8) | 14 (48.3) | 0.834 |
| QI 9. Appropriate duration of intravenous antibiotic treatment should be at least 14 days for uncomplicated SAB, or at least 28 days for complicated SAB | 30 (76.9) | 14 (48.3) | 0.021 |
| QI 10. Appropriate dose and dosing interval of antimicrobial  (VCM; AUC 400-600 μg·h/mL, TEIC; trough 20-40 µg/mL or DAP; ≥6mg/kg) | 31 (79.5) | 26 (89.7) | 0.331 |
| QI 11. Intravenous-to-oral switch should not be performed in < 72 hr | 39 (100) | 29 (100) | 1.000 |
| Bundle total score in QI 1-11, *median (IQR)* | 8 ( 8, 10) | 7 (6, 9) | 0.004 |
